# On the protective capacity of a safety vest for the thoracic injury caused by falling down

**DOI:** 10.1186/s12938-019-0652-3

**Published:** 2019-04-02

**Authors:** Jing Li, Duanduan Chen, Xiaoying Tang, Hanjun Li

**Affiliations:** School of Life Science, Beijing Institute of Technology, 5 South Zhongguancun Street, Beijing, 100081 People’s Republic of China

**Keywords:** Finite element method, Elderly, Fall, Safety vest

## Abstract

**Background:**

Aged people all over the world are prone to fall down accidentally and be injured with fracture, such as the rib fracture. To protect the elderly, the safety vest has been developed to protect them from being injured when falling down. To effectively protect the elderly, more analysis on the protective capacity of a safety vest under different situation are needed.

**Results:**

Herein, a finite element model based on the computed tomography CT scanning data of a Chinese old female was built, and then used to simulate the process of falling down at different velocities. Analysis and comparison were done on the maximum shear stress, kinetic energy curves and internal energy curves with and without safety vest. The maximum shear stress indicated that the Abbreviated Injury Scale (AIS) 2+ injury risks of rib were 8%, 100% and 100% at the velocities of 1.5 m/s, 2.0 m/s and 2.5 m/s, respectively. The corresponding risks were lowered to 0%, 0% and 60% by the vest, respectively. Furthermore, the vest could absorb the internal energy resulted by the deformation of the thoracic osseous tissue by about 20%, thus decreasing the shear stress and the injury risk.

**Conclusion:**

It is concluded that the safety vest decreases the injury risk when the elderly fall down, thus protects them from being injured.

## Introduction

Population aging has been a common problem all over the world. In 2008, the portion of the population older than 65 in the world was about 13%, and by 2056, it is predicted to be 24–25% [1]. By the end of the 21 century, in every three people in the western world there would be one older than 65 [2]. In China, the portion of the elderly has increased sustainably from 7.0% in 2000 to 10.8% in 2016 [3]. Because of the functionality degeneration of the body, the elderly are prone to fall down, which is defined as “unexpected events in which the participant unintentionally comes to rest on the ground, floor, or lower level” [4]. Campbell [5] found that about one-third of the elderly population older than 65 fall each year, and one half of those older than 85. During all the circumstances where the elderly fall down, falling down indoor occurs the most commonly [6]. Based on the fact that women are easier to fall down in the indoor situation, the women population are more common to become the objective when researching the topic of the falling down of the elderly [5, 7–9, 11]. Besides, occurrences of falling down of women are more than those of men for all age groups [10], and a research on the fracture reported that the incidence of fracture is greater among women [11].

Since falling down in the elderly usually results in bad consequence, they are afraid and restricted to do exercise, lowering their life of quality. More seriously, they might suffer from injury or death by falling down accidently [12–14]. Hayes [15] and Elkington [16] reported that injury caused by falling down is the most common and most serious medical care problem for the elderly. According to Rubenstein [17], about 10% of the elderly falling down results in bone fracture, among which the rib fracture is a very common situation.

There have been several researches working on developing devices to protect the elderly from being injured when falling down [18–21]. Almost all of these devices are protective airbags with a system to detect the falling action through the sudden variation of the accelerate [19]. When the falling action is detected, the airbag can be quickly filled with compressed air and inflated within about 200–300 ms before the elderly fall down onto the ground. However, the current using algorithms are difficult to recognize the “falling” action precisely, and moreover, it is not convenient for the airbag to be reused. Especially, the research on protection of the thorax of the elderly when falling down has never been reported.

In the equestrian sports the riders can fall occasionally, and for the riders older than 50, the most frequent injury was rib fractures [22, 23]. To provide the riders a good protection, there were several kinds of safety vest designed for them, which has been proved to have good protective capacity in some cases [22]. We considered that the safety vest can potentially have the capacity of protecting the rib of the elderly from being injured when falling down.

The purpose of this study was to assess the risk of the rib fracture for the Chinese elderly when falling down, and the protective capacity of the safety vest by a finite element method to better describe the structure of the Chinese population, we constructed a new finite element model of the thoracic osseous tissue based on the computed tomography (CT) scanning data of a Chinese old female, instead of using the available finite element model like the total human model for safety (THUMS). Through our finite element model, the falling process was simulated to assess the risk of the rib fracture. Lastly, through comparing the computed results with and without safety vest, its protective capacity was then analyzed.

## Method

**Fig. 1:**
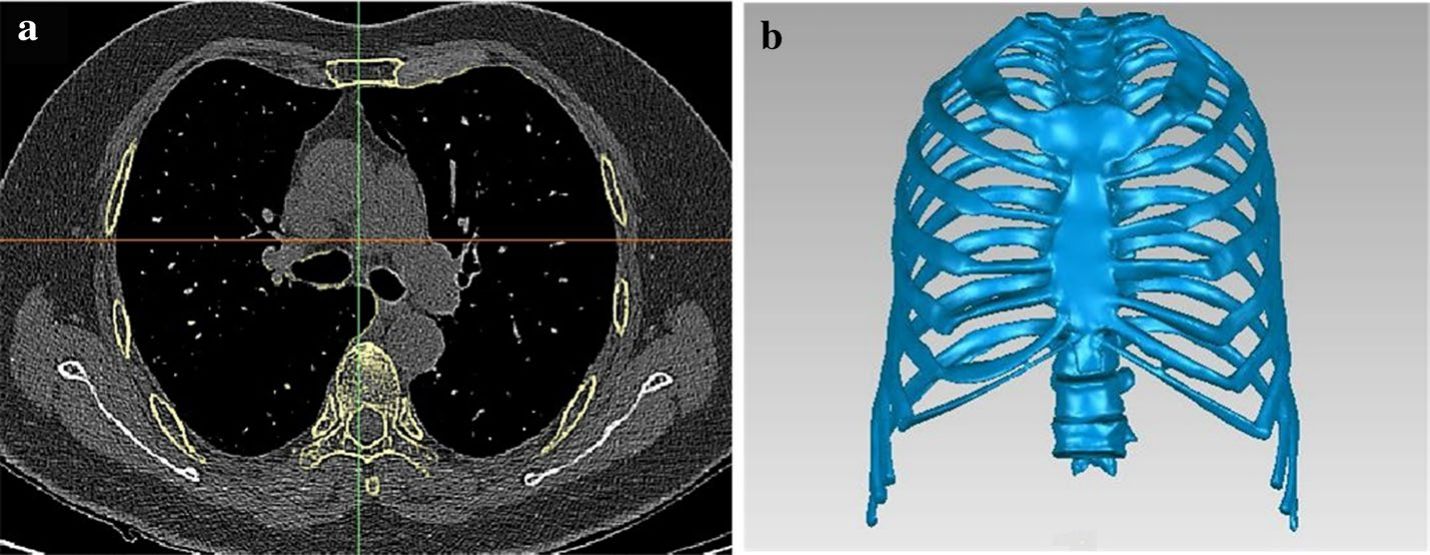
**a** 2D CT image of the thoracic tissue and **b** the final geometry

Due to the fact that more women fell, and the incidence of the fracture is greater among women, we chose the CT scanning data from a Chinese female to construct our finite element model as shown in Fig. 1. Through the CT data, we built the whole thoracic osseous tissue, containing the ribs, sternum, rib cartilage and the thoracic vertebrae. The geometry was built with Mimics (Materialise Inc., Belgium) and Geomagic Studio (Geomagic Inc., USA), the pre-processing was done with HyperMesh (Altair Engineering, USA), all the simulations were performed with the FE solver LS-DYNA (LSTC Inc., USA), and post-processing with LS-PREPOST (LSTC Inc., USA). Any other soft tissues were not considered.

### Creation of the finite element model

The CT scanning data was based on a 65-year-old Chinese female. The slice thickness was 0.620 mm and a total of 545 slices were scanned using a Philips/CT 256 scanner as shown in Fig. 1a. No lesion signals were found in the osseous tissue. The geometry containing the ribs, the rib cartilage, the sternum, the thoracic vertebrae (from L1 to L10) was obtained as shown in Fig. 1b. Some simplifications were done for the cartilage. According to the true anatomy structure of human, the 1st–7th ribs were connected with the sternum directly through the cartilage, while for the 8th–10th ribs they were not connected directly with the cartilage. For simplicity, the cartilage connecting the 8th–10th ribs were discarded. Besides, it is difficult to recognize the CT value of the cartilage precisely, therefore we drew the cartilage according to the anatomy structure. The intervertebral disks were built during the mesh process directly.

Many attempts have been made to use hexahedral elements to mesh the thoracic osseous tissue [24–27]. However, although the hexahedral elements do have better computing accuracy and efficiency, it is difficult and quite time consuming to get a finite element model with good quality only using the hexahedral elements to mesh a complicated and irregular geometry. Furthermore, many of the complex details are not accurately expressed, especially for the human skeleton. Therefore, we preferred the tetrahedral elements, even though there might be a concern that the tetrahedral elements may produce a model with lower computing accuracy and worse computing results. Actually, this concern can now be compensated by employing the rapidly advancing computing capacity. In 1992, Cifuentes found that tetrahedral elements can produce results that are equivalent to those produced with hexahedral elements in both accuracy and CPU time [28].

Each part of the geometry was meshed with tetrahedral elements as the cortical bone. Some researchers used the validated finite element models of the thorax with the element size of 4–5 mm [29–32], using the hexahedral elements. Here we decreased the element size to about 2 mm. For the ribs, sternum and thoracic vertebrae, triangle shell elements of the outer surface were extracted according to the obtained tetrahedral elements as the cancellous bone. For the cartilage we did not distinguish the cortical and the cancellous part, and there was only one component. The mesh of the disks was constructed according to the mesh of the vertebrae, and the elements were organized into two components: the nucleus pulposus and the fibrous ring.

**Fig. 2:**
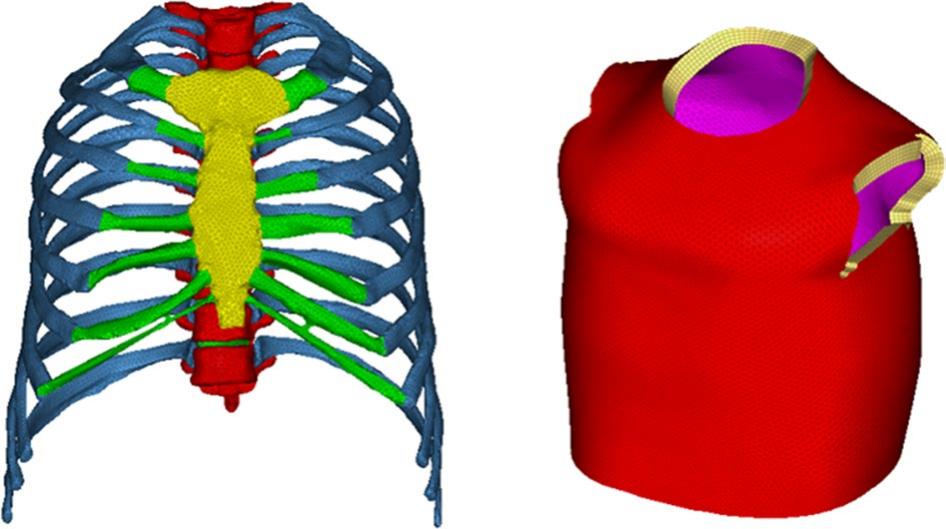
Final mesh of the thorax (left) and the simplified safety vest (right)

A simplified element model of a typical safety vest for horse riders was created [22]. The main of the vest was a foam core with the thickness of 20 mm, and its two surfaces were covered by nylon fabric with the thickness of 0.5 mm. The inner nylon fabric was meshed based on the skin geometry with triangular shell elements. Then they were offset five layers to get the foam core mesh. Lastly the outer surface of the foam mesh was extracted to form the outer nylon fabric mesh. The final mesh of the thorax and the vest was showed in Fig. 2, and the summary of the elements was listed in Table 1. The total element number of the model was 210371 without vest, and 406224 with vest.

**Table 1:** Summary of the elements

Part	Element types	Element number
Rib cortical	Triangular	40454
Rib cancellous	Tetrahedral	64780
Sternum cortical	Triangular	4202
Sternum cancellous	Tetrahedral	11608
Rib cartilage	Tetrahedral	13097
Vertebra cortical	Triangular	22006
Vertebra cancellous	Tetrahedral	51266
Nucleus pulposus	Tetrahedral	1798
Fibrous ring	Tetrahedral	1160
Foam core	Pentahedral	139895
Nylon fabric	Triangular	55958

### Materials

**Fig. 3:**
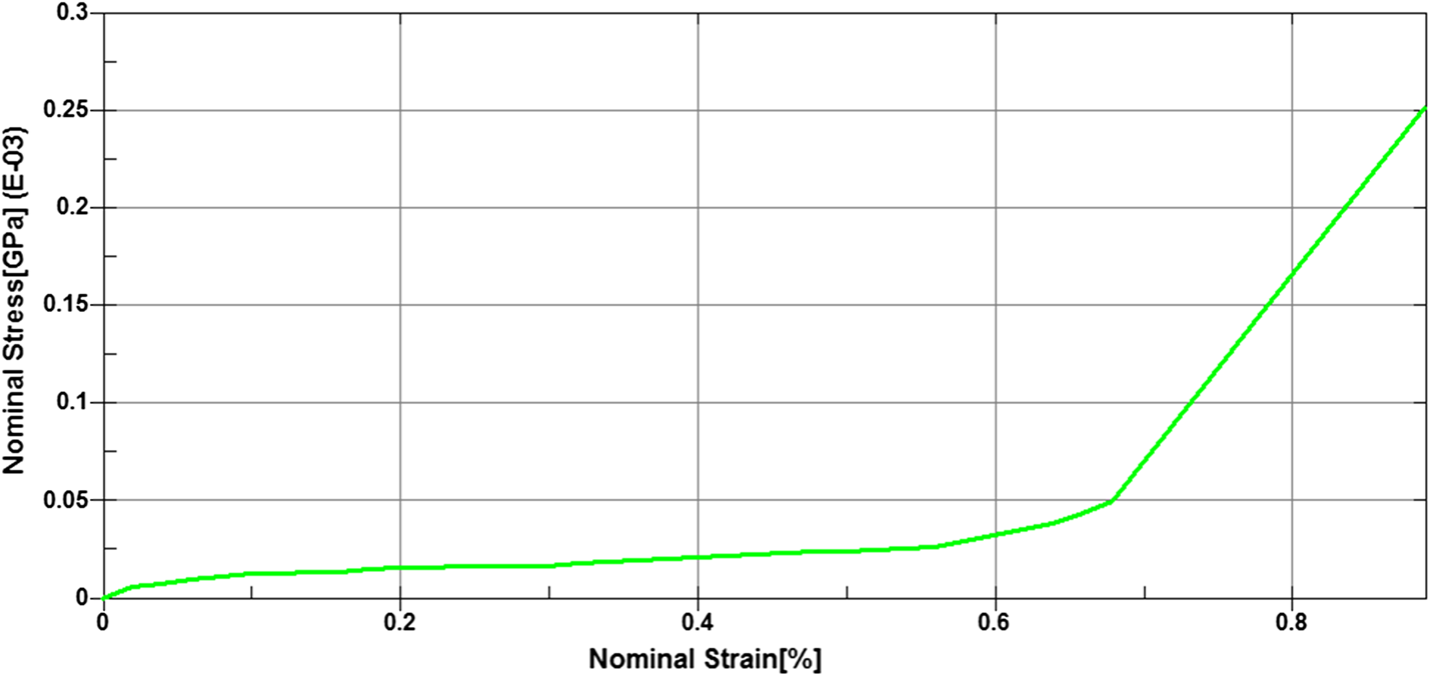
Nominal stress versus strain relationship in the foam core material model

There were some reports on the material model and parameters of the human tissue based on the young cadaver, which has been validated by experiments [33–38]. Here we also adopt these material models directly, all of which are isotropic linear elastic materials. The cortical bone of the elderly is thinner than that of the young generation because of the osteoporosis and some other diseases. Therefore, we assume that the thinner cortical bone is the main cause for the worse bone mechanical properties for the elderly. Other material parameters of cortical and cancellous bone were considered to be the same as the reported. We assign all the cortical bone a constant thickness of 0.6 mm, thinner than the reported value in this study [40]. The foam core and the nylon fabric were simulated with LS-DYNA material model 057 and linear elastic material, respectively. According to literatures [22, 37], a definition of the density and a nominal stress versus nominal strain relationship is enough for the impact model. Failure of the material was not considered. All of the material parameters and the corresponding references were listed in Table 2, and the nominal stress versus nominal strain curve was shown in Fig. 3.

**Table 2:** Material parameters used in the model

Part	Density (kg/m^3^)	Elastic modulus (MPa)	Poisson’s ratio	Stress–strain relationship	Refs.
Rib cortical	1000	40	0.45	Linear	[36]
Rib cancellous	2000	10,000	0.3	Linear	[34]
Sternum cortical	1000	40	0.45	Linear	[34, 36]
Sternum cancellous	2000	10,000	0.3	Linear	[35]
Rib cartilage	1000	49	0.4	Linear	[33]
Vertebra cortical	1000	1000	0.3	Linear	[35]
Vertebra cancellous	2500	11,000	0.4	Linear	[36]
Nucleus pulposus	1040	2300	0.4	Linear	[35]
Fibrous ring	1040	300	0.4	Linear	[35]
Foam core	240	–	–	Figure 3	[37]
Nylon fabric	1000	2000	0.32	Linear	[38]

### Load and the boundary conditions

To evaluate the protective capacity of the vest, the processes of falling down with and without vest at different velocities were simulated and compared, with the anterior part of the thorax impacting onto the floor. According to the literature [39], the velocity of falling can be varied among the range of 1.2–4.79 m/s due to the height and weight. Here three different velocities, 1.5 m/s, 2.0 m/s and 2.5 m/s, were chosen to be the falling velocities. In this study the model was assumed to have only translational velocity along the falling direction, and all the other motion components, such as gravitational acceleration, angular velocity and angular acceleration, were not considered. Therefore, the velocities remained constant prior to the impact with the floor, and the distance between the thorax and floor would not affect the results. The floor was assumed to be a rigid wall when the process of falling down within the indoor circumstance was simulated. The nodes in the boundary of vertebra and disc, sternum and rib were equivalent, so any boundary conditions were not needed. The tied-node-to-surface contact type was used to simulate the joints between ribs and their relating vertebrae. A contact was automatically built when we set a rigid wall and all we need to do was to select all the nodes as the slave nodes. To consider the effect of the whole body during the falling process, the upper nodes of the 1st vertebra and the lower nodes of the 10th vertebra were constrained so that they had only the displacement degree of freedom in the falling direction.

## Results

Six different simulations were accomplished in total, three without vest, and three with vest. During the process of falling down, the thoracic osseous tissue impacted the rigid wall at different velocities, and then rebound.

**Fig. 4:**
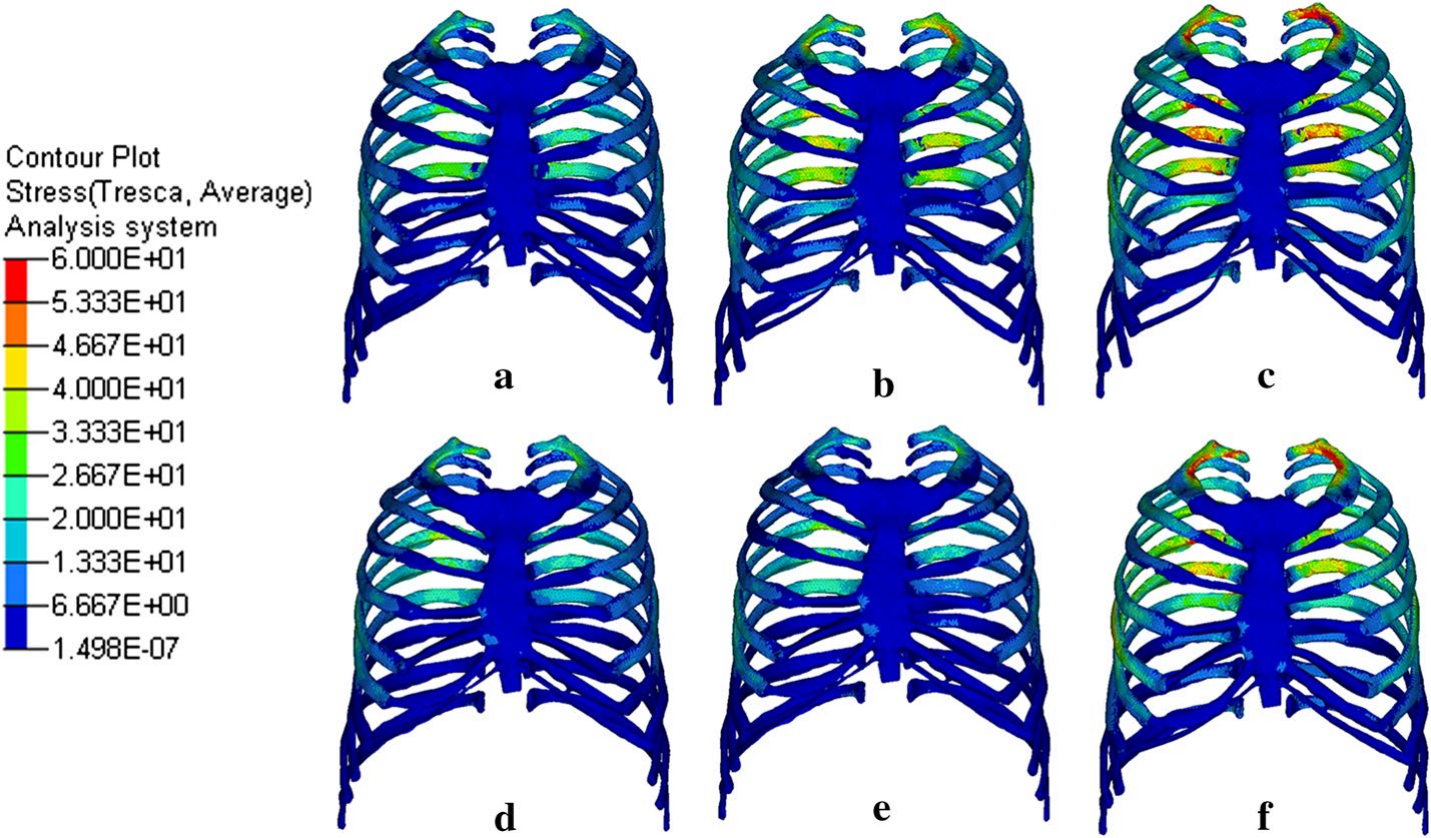
Maximum shear stress plot with and without vest at different velocities. **a** 1.5 m/s, **b** 2.0 m/s and **c** 2.5 m/s without vest, at velocity of **d** 1.5 m/s, **e** 2.0 m/s and **f** 2.5 m/s with vest

Figure 4 showed the maximum shear stress distribution of the rib, sternum and cartilage at different velocities with and without vest. Vertebrae were not included because they were usually not considered when predicting the injury risk of the thorax. During the falling process, the thorax impacted the floor, and the velocity decreased as the thorax deformed. The deformation of the thorax reached the maximum degree when the velocity decreased to 0, at which point we got the maximum stress distribution. Stress is a criterion for predicting the injury risk of the rib under impact loading, and the higher stress means that rib fracture can occur easier. Figure 4a–c show the stress distribution without vest, at velocity of 1.5 m/s, 2.0 m/s and 2.5 m/s, respectively. The time we got the maximum stress plot at the three velocities were 30.8 ms, 26.8 ms and 25.0 ms from the beginning of the calculation. The corresponding maximum shear stress values were 60.59 MPa, 69.20 MPa and 80.85 MPa, respectively. The high stress locations were distributed approximately in the middle of the 1st, 4th–6th rib, where the maximum curvature existed. Figure 4d–f show the stress distribution at three velocities with vest. The time we got the maximum stress plot were 27.8 ms, 24.6 ms and 22.8 ms, and the maximum shear stress values were 48.07 MPa, 55.60 MPa and 66.42 MPa, respectively. When evaluating the injuries of thorax impacting, Abbreviated Injury Scale (AIS) was commonly used to predict the global injury level of the thorax. Mendoza-Vazquez [31] used an Injury Risk–Shear STRESS curve to predict the AIS 2+ injury risk during the frontal thorax crashing according to the maximum shear stress. It is a very steep curve and the risk can increase from 0 to 100% during a very narrow interval around 60 MPa. According to this criterion, the AIS 2+ injury risks of the ribs at velocities of 1.5 m/s, 2.0 m/s and 2.5 m/s were 8%, 100%, 100% without vest, and 0%, 0%, 60% with vest. It is obvious that the injury risk has been reduced with the protection of the safety vest.

**Fig. 5:**
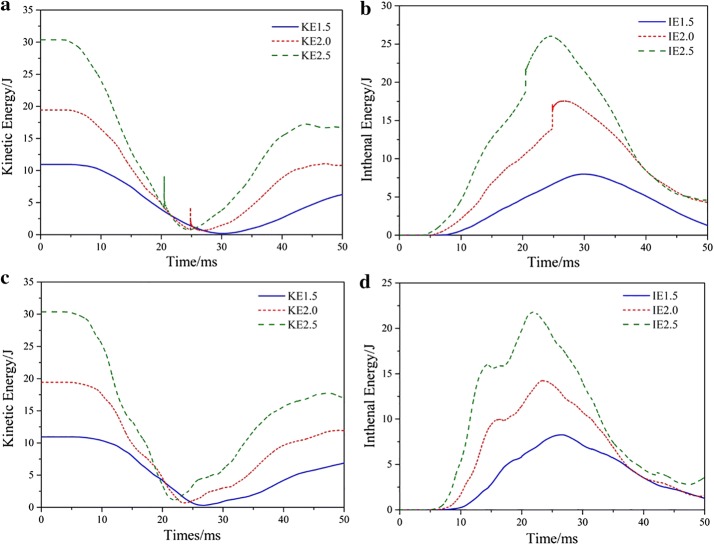
Kinetic energy curves and Internal energy curves with and without vest at different velocities. **a** The KE curves and **b** IE curves for the models without vest, and **c** KE curves and **d** IE curves for the models with vest

Figure 5 shows the kinetic energy (KE) curves and the internal energy (IE) curves of the thoracic osseous component from time 0 to 50 ms. Figure 5a, b were the KE curves and IE curves for the models without vest, Fig. 5c, d were the KE curves and IE curves for the models with vest. The KE remained constant before the thorax contacted the rigid wall with the maximum values. For cases without vest, the values were 10.931 J, 19.434 J and 30.365 J as the velocity increased. For the cases with vest, the KE values were the same as those without the vest corresponding to the three velocities.

After that, the KE began to decrease. Then, as the rebound of the system began, the KE values increased slowly. The minimum KE values were nearly zero, for instances without vest, the values were 0.194 J, 0.652 J and 0.761 J as the initial velocity increased. For instances with vest, they were 0.305 J, 0.697 J and 1.146 J. After that, as the system rebounded, the KE began to increase It is predicted that the rebound velocity would reach an approximate constant value lower than the initial value.

The change of the IE values was just the opposite. At the beginning, the IE values were zero, and they began to increase when the contact between the system and the rigid wall occurred. At the maximum stress point, they reach the maximum value and then decreased. The maximum IE values for instances without vest were 7.981 J, 17.564 J and 26.032 J. As the vest was on, they were lowered to 6.271 J, 14.244 J and 21.788 J, respectively.

## Discussion

During the biomechanical simulation of the human body, it is not realistic to describe material model and its parameters accurately. Therefore in simulations where fractures were possibly to occur. A better method was to assess the fracture and injury risk according to the stress distributions and deformations, rather than considering the fitted failure criterion. The maximum shear stress is an important factor when assessing the rib injury under impact [22, 31]. In this study the AIS 2+ injury risks were obtained according to the maximum shear stress, and then compared to evaluate the protective of the safety vest. Through the results, we could conclude that the maximum shear stress in the thoracic osseous tissue was lowered by wearing a safety vest when falling down. There was a low risk of rib fracture even without vest when the falling velocity was lower than 1.5 m/s. As the velocity increased, the risk increased rapidly and wearing the vest can significantly reduce the injury risk to a very low level to protect the target from being injured. However, the risk of injury remained at high level (60%) even with the safety vest when the velocity is higher than 2.5 m/s.

The high stress zones were mainly at the 1st, 4th–6th ribs where it is much easier to occur rib fractures. Li [40] found that fracture occurred more commonly in the 4th and 6th ribs through experiments and simulations, and the locations corresponded to the maximum curvature points. The lung and the heart would be damaged and lead to other serious injury when the 4th–6th ribs fractured. Furthermore, they compared and studied the effect of the rib cortical thickness on the fracture locations [40]. In reality, the cortical thickness of rib varied along the rib, and different human body had different cortical thickness [41, 42]. They found that although different thickness indicated different stress and the risk possibility are different. However, the fracture locations were the same even the rib were with different thickness. Therefore, we used a constant cortical thickness in the models. Future work will be needed to find an optimized thickness.

The influence of the vest on the value of KE was very limited, but that on the IE values was relatively significant. The IE indicated the elastic deformation resulted from the impact. Thanks to the vest, the IE value increased more slowly, and the maximum IE value at the three velocities were lowered by 22.42%, 18.90% and 16.30%, respectively. The decreased part was absorbed by the vest. Because the IE value indicated the deformation extent and thus the stress level, lower IE value means lower injury risk. We noted that the curve reaches a constant value rather than 0. It is because the motions of the anterior and posterior part of the system were not coherent, and the velocities of the different part of the model will not reach 0 at the same time. Therefore, the minimum value of the KE curve was not 0. In our models it was the elastic deformation and would disappear after an enough long time. But in reality there will be plastic deformation even if no fracture occurred, so this kind of energy difference will still exist and never disappear.

From the curves without vest we could also see that at a relative high velocity (2.0 m/s and 2.5 m/s), there was a sudden “jump” in a short time before the maximum or minimum value reached. This might be the vibration caused by the contact between the bone and the rigid wall. This situation was not found at the low velocity of 1.5 m/s. Furthermore, the vest could be a buffer between the bone and the rigid wall and thus eliminate this vibration when it was on. In computing dynamics, it is possible to be unstable when a rigid body contact with a relative “soft” body at a high velocity. The vest was proven to be able to avoid this problem, but we still do not know whether this is valid at a higher velocity. Therefore, we think that avoiding the contact with rigid body could improve our results. Of course, the results we obtained now were still good enough under the reasonable simplifications.

In conclusion, the vest does have the capacity of decreasing the injury risk when the elderly fall down. From the stress distribution we can find that the stress is localized. To improve the protective capacity of the vest, we can increase the thickness of the locations relating to the high stress, and decrease the thickness at the low stress location to make the vest more flexible.

It should be noted that the results we got in this work could be used to predict the protective capacity of the safety vest even some simplifications have been used in our model. Further work will be done to optimize our model.

There were several limitations that would affect the final results in this study. Firstly, to simulate the falling down process of a person, there were many factors should be considered, such as the body weight, accelerate and the angular accelerate caused by the gravity. In this study we simplified the models to have only the translation degree of freedom and a constant initial velocity, the other factors mentioned were not considered. In our future work we will consider these factors in our models progressively. Secondly, our models were constructed according to one single Chinese old female. Therefore, to be more accurate, more comparisons based on different objectives containing different properties are needed. Thirdly, during the falling process the organs and other soft tissues within the torso can also serve as the buffer, and therefore decrease the stress on the rib. We planned to add these soft tissue to improve the models. Lastly, the tied contact between the rib and the vertebra was just the approximate of the truth, leading to the virtual high stress at this location. Using better method to describe the joint will improve the result.

## Conclusions

In this study we constructed the finite element model based on the CT scanning data of a Chinese old female, and then used this model to simulate the process of falling down at different velocities. Analysis and comparisons were done between the results with and without a safety vest. The maximum shear stress indicated that the AIS2+ injury risks of rib were 8%, 100% and 100% at the velocities of 1.5 m/s, 2.0 m/s and 2.5 m/s. Those risks were lowered to 0%, 0% and 60%, respectively by the protection from a safety vest. Furthermore, the vest could absorb the internal energy resulted from the deformation of the thoracic osseous tissue by about 20%, thus reducing the shear stress and the injury risk. It is believed that the safety vest could decrease the injury risk when the elderly fall down, and thus protect them from being injured. The result provided a way of studying how to protect the Chinese elderly during the process of falling down.
